# Activities of plasma indoleamine-2, 3-dioxygenase (IDO) enzyme in Nigerian patients with lung diseases: *basis for tryptophan supplementation or IDO inhibitor use*

**DOI:** 10.1186/s43168-022-00174-2

**Published:** 2023-01-09

**Authors:** Ganiyu Olatunbosun Arinola, Issa Abdullahi, Sheu Kadiri Rahamon, Zainab Bolanle Fasasi, Olajumoke Oluwaseun Adedeji, Adigun Kehinde, Adekunle Akeem Bakare

**Affiliations:** 1 Department of Immunology, University of Ibadan and University College Hospital, Ibadan, Nigeria; 2 Department of Zoology, University of Ibadan, Ibadan, Nigeria; 3 Biomedical Engineering Department, First Technical University, Ibadan, Nigeria; 4 Department of Family Medicine, University College Hospital, Ibadan, Nigeria

**Keywords:** Asthma, COVID-19, Indoleamine-2, 3-Dioxygenase, Pulmonary tuberculosis, Tryptophan

## Abstract

**Background:**

Clinical trial of IDO inhibitor or uses of micro-nutrient supplements during management of diseases is commonly done without having adequate basis for the practise. Tryptophan (Trp) is an essential amino acid needed for T-lymphocyte function, and indoleamine-2,3-dioxygenase (IDO) is a potent immunoregulatory molecule that catalyses the rate-limiting step of Trp degradation in the kynurenine (Kyn) pathway.

**Materials and methods:**

Human IDO in the plasma samples was measured using ELISA in patients with non-infectious (asthma) and infectious diseases (pulmonary tuberculosis and COVID-19) compared with corresponding un-infected controls.

**Results:**

Mean IDO activity in COVID-19 patients was significantly higher compared with corresponding control (*p* = 0.001) while mean IDO activity in pulmonary tuberculosis patients was non-significantly higher compared with corresponding control (*p* = 0.520), and mean IDO activity in asthma patients was non-significantly lower compared with corresponding control (*p* = 0.102).

**Conclusion:**

Our data suggest that IDO activity as an innate immune factor is increased in infectious lung diseases (COVID-19 and pulmonary tuberculosis) but reduced in non-infectious disease (asthma) and that use of tryptophan supplementation or IDO inhibitor may not be necessary in all lung diseases.

## Background

Pathogens associated with human diseases have evolved multiple sophisticated strategies to avoid immune destruction such as elicitation of oxidative burst, delayed phagosome maturation, and inhibition of IFN-γ signaling [1] and suppressed or delayed production of activating cytokines [2]. However, one mechanism by which phagocytes exert their immune-regulatory ability is through regulation of tryptophan catabolism pathway wherein indoleamine 2, 3-dioxygenase (IDO) converts tryptophan to kynurenine [3]. Thus, IDO-producing phagocytes deplete local tryptophan levels in T-lymphocytes [4] so as to inhibit T cell proliferation, promote T cell death, and skewing Th1/Th2 balance toward Th2 bias [5, 6].

The immunoregulatory molecule IDO has been shown to play a critical role in various pathological conditions, including pregnancy, cancer, and infectious diseases [7–9]. Over-expression of IDO by cancer cells is itself a fundamental immune escape mechanism, and higher IDO activity is a novel prognostic factor in several types of cancer patients [10, 11]. Similar to neoplasm, higher serum IDO activity was found to be a prognostic factor in patients with bacteremia and sepsis [12]. However, in infectious diseases, the precise role of IDO is not yet fully determined and the importance of IDO during infectious disease events is only beginning to emerge. Originally, IDO was considered to function as an antimicrobial molecule, but it also exerts a potent immunosuppressive and immune-tolerance effect. Thus, it is unclear whether increased IDO activity in infections is beneficial for host defense. To date, several studies have examined the effect of blockading IDO in animal models of infection [11, 13]. Bozza et al. [13] demonstrated that IDO inhibition exacerbated *Candida albicans* infection, while Jung et al. demonstrated that blockade of IDO activity protected mice against lipopolysaccharide (LPS)-induced endotoxin shock [14]. In *Mycobacterium avium* infection, blockading IDO reduced the antimycobacterial effect of immunostimulatory oligodeoxynucleotide analogs in vitro [15].

Previous studies on *Toxoplasma* spp and β-Streptococci revealed that IDO is able to deprive an invading organism of tryptophan, thereby halting their replication [16, 17]. It has also been shown that IDO is induced as a result of infection with HIV [18], influenza [19], hepatitis C [20], and *Plasmodium yoelii* [21]. More so, it has been reported that *Leishmania major*-induced IDO expression reduces T cell proliferation and attenuates pro-inflammatory responses in a murine model system [22]. Donovan et al. [23] showed that one of the mechanisms behind the establishment of *Leishmania* spp infection is through the induction of dendritic cell-expressed IDO. However, there have been few studies focusing on IDO activities in pulmonary tuberculosis (PTB), COVID-19, and asthma. Almeida et al. [24] reported that mRNA expression of IDO in induced sputum cells was increased in PTB patients and decreased after anti-TB treatment. Li et al. [8] suggested that IDO is responsible for the impairment of T cell functions in TB pleurisy. In COVID-19 patients, IDO activities was reportedly raised [25, 26] while in asthma patients, IDO activities was reported to be reduced [27, 28].

Pro-inflammatory activity was reported as the main stimulus for the conversion of tryptophan to kynurenine and IDO activation [7, 13]. Also, previous studies have shown that bacterial and viral products can induce IDO expression in a variety of cell types, including monocytes/macrophages, dendritic cells, and endothelial cells [3, 4, 11, 24]. Likewise, in PTB, these cells are involved in immune responses during COVID-19 and asthma episodes. It is thus hypothesized that the activities of IDO will vary in patients with asthma, tuberculosis, and COVID-19 considering varied degrees and mechanisms of inflammation in these different diseases. We aimed to investigate the discriminatory potential of IDO activity during different non-severe lung diseases, thus determining the relevance of tryptophan supplementation in these groups of patients. Our results suggest that not all diseases require essential amino acid tryptophan supplementation during management and that IDI inhibitor will not be beneficial in every disease.

### Subjects and methods

The study protocol was reviewed and approved by the University of Ibadan/University College Hospital Joint Institutional Research Ethics Committee (Approval number is UI/EC/20/0233), and informed consent was obtained from the participants. Newly diagnosed, non-severe and stable pulmonary tuberculosis patients, newly diagnosed COVID-19 patients, and newly diagnosed asthma patients were recruited from the University College Hospital, Ibadan, Nigeria, for this quasi-experimental study. Corresponding sex and age-matched control subjects were recruited from staff and students of the University College Hospital, Ibadan, Nigeria.

Five milliliter of blood was withdrawn from the antecubital fosa vein into lithium heparin. Plasma was obtained from the blood after centrifugation of the whole blood at 4000 rpm for 10 min. Human indoleamine 2, 3-dioxygenase in the plasma samples was measured using ELISA as described by the manufacturer (E-EL-H2162, Elabscience Biotechnology, China). Absorbance was measured at 450nm with an ELISA reader (Spectra Max Plus 384 Molecular Devices LLC, USA). Student’s *t* test was used to compare the mean values. The *p* value ≤ 0.05 was considered as statistically significant.

## Results

The mean ± S. D of IDO activities in Nigerian patients with asthma, pulmonary tuberculosis, and COVID-19 compared with corresponding controls is presented in Table 1. The result in Table 1 shows that mean IDO activity in COVID-19 patients was significantly higher compared with corresponding control (*p* = 0.001). Also from the same table, mean IDO activity in tuberculosis patients was non-significantly higher compared with corresponding control (*p* = 0.520) and mean IDO activity in asthma patients was non-significantly lower compared with corresponding control (*p* = 0.102).

**Table 1:** The mean + S. D of plasma IDO activities in Nigerian patients with asthma, pulmonary tuberculosis, and COVID-19 compared with controls

Parameter	Tuberculosis (*n* =30 )	Control (*n* =30 )	*t* values	*p* values
IDO (ng/ml)	19.17 ± 4.35	18.35 ±2.62	0.65	0.52
	COVID-19 (*n* = 20)	Control (*n* = 20)		
IDO (ng/ml)	22.40±2.54	18.40±2.72	4.82	<0.001*
	Asthma (*n* = 15)	Control (*n* = 15)		
IDO (ng/ml)	19.70± 2.96	22.00±4.15	1.69	0.102

## Discussion

Tryptophan is an essential amino acid which cannot be newly synthesised but has to be taken in diet, and thus is of limited availability to humans. Human T cells, once stimulated to proliferate, essentially depend on the availability of tryptophan to synthesize immune-active proteins. Tryptophan breakdown occurs mainly along the kynurenine pathway using two enzymes such as TDO (tryptophan 2, 3-dioxygenase) whose activity is restricted to the liver and IDO (indoleamine 2,3-dioxygenase), which is active throughout the body [29]. This raises the importance of immune-regulatory activities of IDO in disease states. IDO regulates immune responses by alteration of Trp availability in intercellular microenvironment where T cells encounter antigen. T cells themselves have not been observed to express IDO [27, 29], but IDO expression and activity have thus far been demonstrated in multiple cell types, among which are bronchial epithelial cell, eosinophils, monocytes, macrophages, and the dendritic cells [27, 29, 30]. These antigen-presenting cell populations are important in TB, COVID-19, and asthma patients, thus supporting the basis for the determination of IDO activities in these groups of patients.

Indoleamine 2,3-dioxygenase is the rate limiting enzyme in Trp catabolism is ubiquitously distributed in various organs of mammals including the brain, lung, spleen, and alimentary tract [28]. The enzyme activity in various tissues of human subjects has a considerable individual variation in the activity of each tissue; the activity was relatively high in the lung and small intestine, moderate in the spleen and stomach, and low in the other tissues [28], thus explaining why IDO activity was significantly higher in COVID-19 patients, a multi-organ disease [31] compared with its control. The enzyme IDO (indoleamine 2, 3-dioxygenase) inhibits the proliferation of intracellular pathogens by depriving them of essential tryptophan [4]. Evidences indicate that this enzyme functions as an antimicrobial defense mechanism by allowing cells to deplete tryptophan from intracellular pool or local microenvironment [3, 4]; thus, the activity is expected to be high during intracellular infections. Since asthma is not an infectious disease, hence the reduced IDO levels observed in our asthma patients compared with corresponding control.

The immunoregulatory enzyme IDO controls tryptophan metabolism and is induced by pro-inflammatory stimuli and in diseases that are associated with chronic stimulation of Th1-mediated immunity [7, 13]. The most potent inducer is IFN-gamma although other inflammatory stimuli, including type-1 interferons (IFNs) (IFNs-α and β), bacterial lipopolysachharides, some viruses, and intracellular pathogens, can induce expression of IDO gene [11, 24]. IFN gamma was shown to be highly expressed in COVID-19 patients [2, 25, 26] and PTB patients [32]; therefore, IDO activity is expected to be high in COVID-19 patients as found in this study. In infectious diseases, IDO is an antimicrobial molecule acting through local starvation of Trp, which is an essential amino acid for bacterial growth. By reducing the local Trp concentration and producing immunomodulatory Trp metabolites, such as Kyn [16–23], IDO potently inhibits T cell functions and generates regulatory T cells (Treg), leading to immune suppression or tolerance [5, 6]. We observed a slight increase in mean plasma IDO in PTB patients. Raised IDO level results in increased breakdown of tryptophan, depletes intracellular level or availability of tryptophan for *Mycobacterium tuberculosis* proliferation, at the same time reduce T cell functions and increasing susceptibility to infections.

Allergy involves eosinophilia and Th2 polarization while indoleamine 2, 3-dioxygenase (IDO) regulates T cell functions. Human eosinophils constitutively express IDO and eosinophils infiltrate the lungs of asthma patients and associated lymphoid tissue to exhibit intracellular IDO immunoreactivity locally [29]; thus, the blood level of IDO activity might be low in asthma patients as reported in this study. A study also reported that IDO-1 enzyme activity was lower in patients with asthma and allergic rhinitis than in controls [27]. Another previous study revealed that atopy was associated with low IDO-1 activity [30], and serum tryptophan level was found to be higher in patients with pollen allergy than in healthy blood donors [28], possibly because of suppression of IDO-1 enzyme activity. Maneechotesuwan et al. [31] reported low IDO activity has previously been observed in atopy and asthma. IDO can promote the generation of various toxic Trp metabolites, which induce Th cell death [5, 6]; therefore, reduced IDO in asthma might be to inhibit Th2-mediated asthma and to attenuate Th1-mediated pulmonary inflammation. A similar mechanism has been observed in the placenta, where high tissue levels of IDO inhibit maternal T cell responses against the developing fetus [9].

Infection with severe acute respiratory syndrome coronavirus 2 (SARS-CoV-2) causes coronavirus disease 2019 (COVID-19) which is accompanied by activated immune-inflammatory pathways and oxidative stress [2], both induces IDO production [7, 13]. Our results indicate increased activity of the IDO enzyme in COVID-19. Almulla et al. [33] reported similar result and also implicated IDO in the pathophysiology, complications, and progression of COVID-19. Some COVID-19 patients may experience acute respiratory distress or even severe acute respiratory syndrome, organ failure, and death, especially in older people and people with type 2 diabetes mellitus (T2DM), high blood pressure, heart disease, stroke, dementia, and obesity [2]. COVID-19 is characterized by activated immune-inflammatory pathways and, in some cases, hyperinflammation [2]. Profound tissue damage, even extending to organ failure coupled with hyperinflammation, increased production of reactive oxygen species (ROS), and oxidative damage associated with COVID-19 might contribute to significantly high level of IDO in COVID-19 patients considered for this study.

Nitric oxide production can inhibit IDO expression leading to high tryptophan levels [34]. The level of plasma NO was reduced in tuberculosis patients [35], but the level of plasma NO was reportedly high in asthma patients [36]. These previous reports of plasma NO in tuberculosis and asthma patients from the same environment support the IDO result of the present study. IDO activation has been shown to increase TRYCATs such as kynurenine (KYN), 3-OH-kynurenine (3HK), kynurenic acid (KA), quinolinic acid (QA), and xanthurenic acid (XA) but lowered Trp level. Some TRYCAT neutralize ROS, have antioxidant properties, decrease IFN-γ production, increase IL-10 production [37], and have neuroprotective effects [38]. Secondly, reduced TRP exerts anti-inflammatory (reduced T cell proliferation and activation, sensitization of apoptosis of activated T cells, and induction of the regulatory phenotype) and antimicrobial (inhibiting the growth of viruses, bacteria, and parasites) effects through Trp starvation [4, 21–23]. Thus, increased IDO in our COVID-19 patients might be among innate factors protecting COVID-19 patients, reducing severity, and accompanied complications. Though some TRYCATs including KYN, 3HA, 3HK, picolinic acid (PA), XA, and QA have depressogenic, anxiogenic, and neurotoxic effects [38], it could exacerbate the neuro-immune and neuro-oxidative toxicity, resulting in comorbid affective disorders [39]. Therefore, it is safe to say that the raised IDO activity in SARS-CoV2-infected patients may play a role in the neuropsychiatric and cognitive syndromes of long or post-COVID syndrome. It will therefore be advisable to determine IDO in relation to severity of COVID-19 or patients at admission and discharge.

It may be hypothesized that COVID-19-associated IDO activation may aggravate the existing disorders in this pathway in comorbid disorders (obesity, dementia, T2DM, hypertension and heart disease, stroke, chronic obstructive pulmonary disease (COPD), and chronic kidney disease) [2]. Indeed, in all those comorbid diseases, the IDO enzyme was activated as indicated by an increased KYN/TRP ratio [39–41]. By inference, when COVID-19 develops in people with those comorbid illnesses, an amplified IDO activity response may occur, contributing to aggravated toxicity in addition to the consequences of inflammation and oxidative stress. Preclinical data suggest that IDO inhibition can delay tumor growth, enhance dendritic cell vaccines, and synergize with chemotherapy through immune-mediated mechanisms, and the lead IDO inhibitor, D-1-methyl-tryptophan (D-1-MT), was selected for phase I trials and seems to have immune modulating activity [42]. Li et al. demonstrated that an inhibitor of IDO reversed the depletion T cell-derived cytokine production in the pleural fluid of patients with TB pleurisy [8]. These shows that the IDO pathway is an important mechanism of COVID-19, and blocking it could improve treatment outcomes. Clinical development of D-1-MT and other IDO inhibitors as systemic immunomodulators to be combined with other immune modulators, vaccines, and chemotherapy should be attempted. Inhibition of IDO by 1-methyl tryptophan, a tryptophan analog, resulted in reversal of immune tolerance and suppression.

## Conclusion

Our data suggest that IDO activity as an innate immune factor is increased in infectious lung diseases (COVID-19 and pulmonary tuberculosis) but reduced in non-infectious disease (asthma) and that use of tryptophan supplementation or IDO inhibitor may not be necessary in all lung diseases.

## Data Availability

All data generated or analyzed during this study are included in this published article.
